# Antidepressant-Induced Psychosis: A non-common Case Report

**DOI:** 10.1192/j.eurpsy.2025.2209

**Published:** 2025-08-26

**Authors:** S. Castelao-Almodovar, A. Arce de la Riva, R. Albillos Perez, A. Pérez Balaguer, E. Gil Benito

**Affiliations:** 1 Centro de Salud Mental El Escorial, El Escorial; 2 H. U. Puerta de Hierro, Majadahonda, Spain

## Abstract

**Introduction:**

The emergence of psychotic symptoms induced by antidepressants is an uncommon phenomenon, though it has been documented in isolated cases. Psychosis induced by serotonin-norepinephrine reuptake inhibitors (SNRIs), such as Venlafaxine, is particularly rare. This case presents a patient who developed psychotic symptoms after starting treatment with Venlafaxine, highlighting his capacity for self-criticism and the egodystonic nature of his delusions.

**Objectives:**

To describe a case of a depressive episode with psychotic symptoms secondary to antidepressant treatment, emphasizing the importance of differential diagnosis, therapeutic management, and the patient’s notable awareness of the unreality of his psychotic symptoms.

**Methods:**

A 40-year-old male with a history of depressive disorder and substance abuse experienced high levels of anxiety following the death of his father, with whom he had a conflicted relationship. He started treatment with Venlafaxine, which he had previously taken with good results. Shortly after, he developed euphoria, persecutory thoughts, and delusions, such as the belief that there were cameras watching him, that his food was poisoned, and that he was being followed. No substance use was reported during this period, although he had a history of significant abuse in the past. Due to the worsening of his symptoms, he voluntarily admitted himself for further evaluation at a hospital in Barcelona.

**Results:**

During his hospital stay, Venlafaxine was discontinued due to its association with the psychotic symptoms. Antipsychotics such as Olanzapine, Invega, Aripiprazole, and Depakine were introduced, but these were poorly tolerated. After being transferred to Madrid, Cariprazine was reintroduced, leading to partial improvement, although referential thinking persisted. In private follow-up care, Anafranil was later added, which further improved his mood, although residual psychotic symptoms, particularly referential thinking, remained. A key aspect of this case is the patient’s good insight and egodystonic experience of his psychotic symptoms from the onset. He has recently started group therapy in a Multifamily Psychotherapy Group.

**Conclusions:**

This case highlights the importance of differential diagnosis between antidepressant-induced psychosis and primary psychotic disorders. It also underscores the patient’s egodystonic experience of his delusions, with good insight, which facilitated clinical management. The literature on antidepressant-induced psychosis, particularly with drugs like Venlafaxine, is limited, indicating the need for further study of this rare but significant side effect.

**Disclosure of Interest:**

None Declared

